# Environmental Roots of Asthma

**DOI:** 10.1289/ehp.113-a32

**Published:** 2005-01

**Authors:** M. Nathaniel Mead

From 1980 to 1999, the number of U.S. doctor’s office visits for asthma jumped from about 6 million to nearly 12 million, according to data from the Centers for Disease Control and Prevention, and the World Health Organization estimates cases worldwide at 100–150 million. Epidemiologic studies have linked the disease to a plethora of modern lifestyle factors, but the traditional focus has been on heredity and a few identifiable triggers such as animal dander, fungi, ozone, and pollens. At an October 2004 symposium titled Environmental Influences on the Induction and Incidence of Asthma, cosponsored by the NIEHS and the U.S. Environmental Protection Agency, presenters reviewed the scientific evidence for a wider expanse of predisposing factors, including environmental tobacco smoke (ETS) exposure, obesity, dietary fat intake, oxidative stress, and *in utero* xenobiotic exposures. The emerging array of dynamic interactions between genes, allergens, and pollutants all point to a complex profile of susceptibility and to new possibilities for public health intervention.

## Acquiring a Healthy Tolerance

The critical interactions between genetic susceptibility and environmental exposures in the induction of asthma are likely to be heavily influenced by the developmental phase at which the exposures occur. For example, it has long been suspected that decreased exposure to microbes during early life may be contributing to the rise in asthma incidence. Of pivotal interest, from a developmental perspective, is how, why, and when some people acquire immunological tolerance to common allergens, while others go on to develop asthma.

“The programming of this tolerance begins during prenatal and postnatal development,” said Harald Renz, a research scientist at Germany’s Marburg University. In studies of traditional farm environments in Switzerland, Austria, and Germany, Renz’s team consistently found an inverse relationship between asthma rates and maternal blood levels of the bacterial endotoxin lipopolysaccharide (LPS), which is a marker for exposure to gram-negative bacteria common in farmyards. “Infants born to mothers who maintained their daily farm work during pregnancy were almost completely protected from asthma,” he said. “Animal studies using LPS during gestation have confirmed these findings: the offspring are largely protected against the development of allergic inflammation and respiratory hyperresponsiveness.”

Other investigators urged caution against overly simplistic perspectives on the protective impact of early-life exposures to microbes. “The focus on microbial exposure is only one piece of a much larger picture,” said Peter Sly, a lung specialist at the Telethon Institute for Child Health Research in Perth, Australia. “Throughout early life, the immune system takes maturational cues from the environment in the form of microbial stimulation, bowel flora, mother’s milk, and dietary factors. At the same time, the infant is exposed to allergens in the diet and environment. If the allergen exposure coincides with those normal maturational cues, then you’re less likely to develop allergic sensitization and asthma.”

## An Eye on Inflammation

In western societies, however, said Sly, maturational cues are often missing due to factors such as more sanitary living conditions and use of antibiotics. Problems arise, moreover, when the fetus or infant is exposed to airborne pollutants such as ETS and diesel exhaust particles (DEP), which can cause airway inflammation and may enhance allergic sensitization and drive disease expression. Increased protection against asthma therefore stems from a confluence of early-life microbial exposure, normal immune maturation, and low exposure to airway irritants or inflammatory factors.

The concept of synergy was a repeated theme at the symposium. One prominent example was the interaction between ragweed pollens and DEP, which have received increasing attention as culprits in the rising incidence of asthma. “Many studies have found that DEP enhances airway responsiveness in asthmatics,” said clinical immunologist Andre Nel of the University of California Medical School in Los Angeles. “We also know that DEP has an adjuvant effect on the Th2 cytokine responses [specific immune responses that increase allergic tendencies] to ragweed pollens, causing an allergen-specific IgE response in humans and thus greater susceptibility to asthma.”

Nel has studied the quinones, nitrogen oxides, and other pro-oxidative chemicals in DEP. These chemicals tend to increase oxidative stress and stimulate inflammatory pathways that, in turn, pave the way for asthma. Conversely, thiol antioxidants have been shown to interfere with the effects of DEP. Research is now needed to determine whether antioxidant treatment may be beneficial for children living along roadways with increased traffic density, where asthma prevalence tends to be higher.

Maritta S. Jaakkola, a senior scientist at the Institute of Occupational Medicine of England’s University of Birmingham, reported on several studies showing a strong relationship between the extent of smoke exposure and asthma. Jaakkola’s research has shown that prenatal, infant, childhood, and adult exposures can all predispose individuals to asthma. In a study in Finland, 8% of asthma cases that started in adulthood were attributable to ETS exposure within the preceding year. “Exposures to ETS in prenatal life, early childhood, and adulthood can all raise the risk of asthma,” said Jaakkola. “For adults, even quite recent exposures can make a difference.”

Different groups of mechanisms seem to be involved: ETS may promote chronic respiratory infections in early life, contributing indirectly to asthma risk. In contrast, ETS-related irritants that inflame the airways may play a stronger role in adult cases of asthma. Obese adults might also be at greater risk, given data presented by Stephanie Shore of the Harvard School of Public Health showing increased inflammatory cytokine levels and airway hyperresponsiveness in these individuals.

Additional discussions focused on the identification of inflammatory markers that can serve as potential indicators of asthma risk. Karin Yeatts, a researcher at the University of North Carolina Center for Environmental Medicine, Asthma, and Lung Biology, spearheaded a study on the effects of different particle sizes and their impact on inflammatory markers in adult asthma patients. Preliminary results indicate that a subgroup of the asthmatic adults had increased levels of inflammatory markers in their lungs in response to increases in ambient concentrations of particles smaller than 2.5 microns. “The levels of particulate matter triggering the upper airway responses were actually lower than those specified by the current national regulations,” said Yeatts. “This suggests that a subgroup of asthmatic adults who show this diverse spectrum of inflammatory cytokines in their blood may be at greater risk where the rest of the population would be relatively safe.”

The symposium yielded a number of suggestions for public health interventions to lower asthma incidence. Among the proposed strategies were “healthy home” design and building remediation to minimize humidity and improve indoor air quality; changes in infection control to curb rising asthma rates in the elderly; increased education on maternal smoking as a preventable risk factor; more green belts in urban areas as pollution buffers; stricter sanctions on emissions from automobiles and diesel engines, mandating diesel particle traps; a large-scale shift away from fossil fuel use; greater efforts to reduce exposures to known sensitizers in the workplace (including a ban on smoking at work); and better public health communication to all high-risk groups.

The public health challenge of asthma will call for a confluence of scientific and policy directives. “Ultimately, in tackling the problem of factors associated with asthma incidence, we are facing a new challenge to understand the complexity in host–environment interactions as well as the practical issues in developing social policy,” said presenter Kevin B. Weiss, a professor of medicine at Northwestern University. “The challenge is great, but the potential for public health impact is even greater.”

## Figures and Tables

**Figure:**
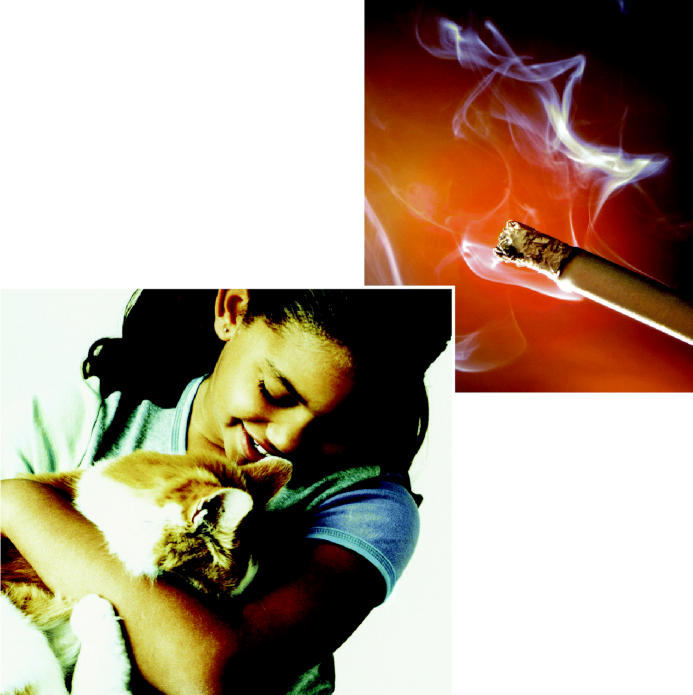
**Cats and clouds.** Animal dander and secondhand tobacco smoke were just two environmental triggers of asthma discussed at a recent meeting cosponsored by the NIEHS and the EPA.

